# Adenosine modifications impede SARS-CoV-2 RNA-dependent RNA transcription

**DOI:** 10.1261/rna.079991.124

**Published:** 2024-09

**Authors:** Laura R. Snyder, Ingrid Kilde, Artem Nemudryi, Blake Wiedenheft, Markos Koutmos, Kristin S. Koutmou

**Affiliations:** 1 Department of Chemistry, University of Michigan, Ann Arbor, Michigan 48109, USA; 2 Program in Chemical Biology, University of Michigan, Ann Arbor, Michigan 48109, USA; 3 Department of Microbiology and Cell Biology, Montana State University, Bozeman, Montana 59717, USA; 4 Department of Biophysics, University of Michigan, Ann Arbor, Michigan 48109, USA

**Keywords:** RNA modifications, RNA viruses, RNA-dependent RNA polymerase, SARS-COV-2, in vitro transcription

## Abstract

SARS-CoV-2, the causative virus of the COVID-19 pandemic, follows SARS and MERS as recent zoonotic coronaviruses causing severe respiratory illness and death in humans. The recurrent impact of zoonotic coronaviruses demands a better understanding of their fundamental molecular biochemistry. Nucleoside modifications, which modulate many steps of the RNA life cycle, have been found in SARS-CoV-2 RNA, although whether they confer a pro- or antiviral effect is unknown. Regardless, the viral RNA-dependent RNA polymerase will encounter these modifications as it transcribes through the viral genomic RNA. We investigated the functional consequences of nucleoside modification on the pre-steady state kinetics of SARS-CoV-2 RNA-dependent RNA transcription using an in vitro reconstituted transcription system with modified RNA templates. Our findings show that *N*^6^-methyladenosine and 2′-*O*-methyladenosine modifications slow the rate of viral transcription at magnitudes specific to each modification, which has the potential to impact SARS-CoV-2 genome maintenance.

## INTRODUCTION

The COVID-19 pandemic brought RNA viruses to the forefront of scientific, medical, and public awareness. Zoonotic coronaviruses had previously received a large research focus, being the cause of the SARS and MERS epidemics (de Wit et al. 2016; Lu et al. 2020). Recently much of this effort has been directed toward understanding the essential activity of SARS-CoV-2 RNA-dependent RNA polymerase (RdRp), nonstructural protein 12 (nsp12), and its inhibition through either the design of novel nucleotide analogs, or the application of preexisting nucleotide analogs such as remdesivir and molnupiravir, as broad-spectrum therapeutic countermeasures (Dangerfield et al. 2020; Jockusch et al. 2020; Shannon et al. 2020a,b; Bravo et al. 2021; Yuan et al. 2021). The ability of the SARS-CoV-2 RdRp to replicate its nearly 30,000 nt genome with sufficient processivity and fidelity is crucial to the viral life cycle. Simultaneously, it must maintain mutational rates that allow the virus to rapidly adapt under evolutionary pressure (Lauring et al. 2013).

Molecular-level analyses of viral transcription are vital to our understanding of the tradeoff between transcription rate and accuracy in the RNA virus life cycle. Emblematic of this, kinetic investigations of SARS-CoV-2 RdRp residues that diverge from consensus coronavirus RdRp sequences reveal an inverse correlation between transcription speed and accuracy (Campagnola et al. 2022). Additionally, single-molecule studies showed that processive transcription elongation follows a fast nucleotide addition pathway, while slow nucleotide addition is associated with polymerase pausing and stalling (Bera et al. 2021). Such findings demonstrate the careful balance between viral RNA transcription speed, processivity, and fidelity, suggesting that any mechanism that disrupts this balance and perturbs SARS-CoV-2 genome maintenance is a potential therapeutic target. Indeed, nucleotide inhibitors of RNA replication have been widely explored as potential treatments for COVID-19. One such example is remdesivir triphosphate, which in vitro transcription studies showed interacts with nsp12 with greater specificity than ATP (Dangerfield et al. 2020; Malone et al. 2023). However, the mechanisms that nature uses to modulate transcription speed and drive viral evolution remain to be established.

**FIGURE 1.**
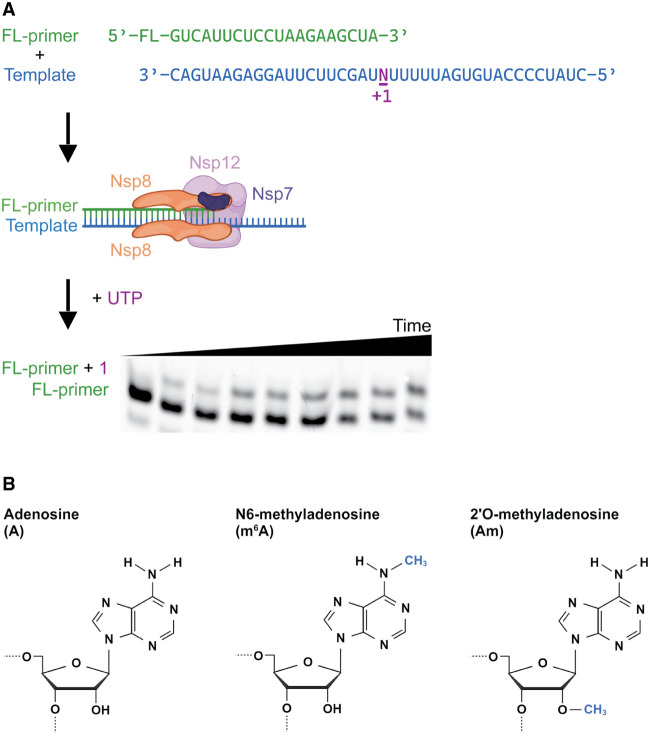
In vitro assay as used to study SARS-CoV-2 transcription on modified RNA templates. (*A*) Experimental workflow of kinetics experiments, showing in order binding of RNA template and fluorescent primer, binding of nonstructural proteins, addition of UTP, quenching at time points, then RNA primer and extension product resolved and visualized on Urea-PAGE. (*B*) Chemical structures of adenosine, *N*^6^-methyladenosine, and 2′-*O*-methyladenosine.

One way that this might plausibly occur is through the epigenetic modification of viral RNA genomes. Indeed, a wide variety of RNA viruses contain posttranscriptional nucleoside modifications on their genomic RNA (Netzband and Pager 2020). Many viruses, including SARS-CoV-2, encode *N*^7^-methyltransferase, and 2′-*O*-methyltransferase proteins in their genome to mimic the eukaryotic 5′ cap structure, evading the host immune system and enhancing translation initiation (Daffis et al. 2010; Hyde and Diamond 2015; Netzband and Pager 2020). Any further modifications would necessarily be incorporated by host proteins (Netzband and Pager 2020; Li and Rana 2022). RNA modifications are known to reduce the immune response to foreign RNA, yet the precise consequences of each individual modification on the viral life cycle are not well established (Netzband and Pager 2020; Li and Rana 2022). Notably, several of the most common RNA modifications, including *N*^6^-methyladenosine (m^6^A), inosine (I), and pseudouridine (Ψ), have been reported in SARS-CoV-2 genomic RNA (Di Giorgio et al. 2020; Kim et al. 2020, 2022; Fleming et al. 2021; Liu et al. 2021; Izadpanah et al. 2022). This has sparked interest in how the viral RNA replication machinery interacts with modified RNA (Jones et al. 2022; Apostle et al. 2023; Petushkov et al. 2023). We sought to understand the role of the nucleoside modifications m^6^A and 2′-*O*-methylation in viral genomic RNA on SARS-CoV-2 RNA-dependent RNA transcription. Multiple m^6^A sites have been identified using both MeRIP-seq and nanopore sequencing, primarily in the 3′ end of the SARS-CoV-2 RNA containing the structural nucleocapsid gene (Burgess et al. 2021; Li et al. 2021). Additionally, 2′-*O*-methylated adenosine (Am) was detected in SARS-CoV-2 RNA by LC–MS/MS nucleoside analysis, which identifies global nucleoside levels but does not report on sequence context (Li et al. 2021). To biochemically characterize these methylated adenosine modifications in the context of SARS-CoV-2 transcription, we conducted kinetic studies of single-nucleotide addition using modified RNA templates using an in vitro reconstituted transcription system (Fig. 1A). Our results demonstrate that m^6^A and 2′-*O*-methylation (Am) (Fig. 1B) impact the rate and processivity of SARS-CoV-2 transcription. Furthermore, we find that the SARS-CoV-2 replication/transcription complex (RTC) can transcribe through these modifications during processive transcription, suggesting that if the RTC encounters modifications in nature, it will likely slow but not terminate transcription. This could provide opportunities for additional cellular factors to act on the viral RNA.

## RESULTS AND DISCUSSION

To examine the impact of RNA modifications on SARS-CoV-2 transcription kinetics, we reconstituted the minimal SARS-CoV-2 RTC. Each RTC contained a single copy of the purified RdRp nsp12, and the copurified subcomplex comprised a single copy of nsp7 and two copies of nsp8 (Supplemental Fig. S1). RTC activity was monitored via the extension of a 5′ fluorescein-labeled primer annealed to a 40 nt template RNA sequence designed from the 3′ end of SARS-CoV-2 genome, resolved by urea-PAGE (Fig. 1A; Supplemental Fig. S2).

**FIGURE 2.**
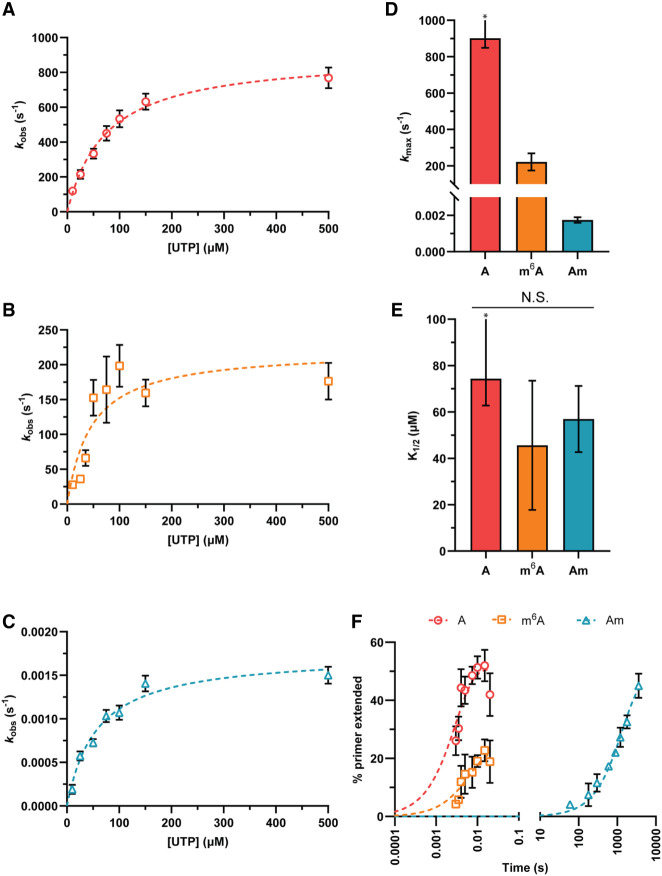
Pseudo-first-order kinetics of SARS-CoV-2 transcription shows that modified RNA templates decrease the rate of nucleotide addition. (*A*–*C*) *K*_1/2_ curves constructed by determining the *k*_obs_ for single-nucleotide addition using RNA templates with adenosine (*A*), m^6^A (*B*), or Am (*C*) in the +1 position over a range of UTP concentrations. (*D*) *k*_max_ values for each template calculated from *K*_1/2_ curve fit. (*E*) *K*_1/2_ values for each template calculated from *K*_1/2_ curve fits. (*F*) Representative time courses for single-nucleotide addition reactions using templates with A, m^6^A, or Am in the +1 position. *The half-time for UTP addition on the unmodified template is lower than the dead time of the quench flow apparatus; therefore, *k*_max_ and *K*_1/2_ represent minimum limits.

We evaluated the impact of having a modified nucleotide in the first position of the template to be decoded by the RTC (+1) (Fig. 2). Following the addition of uridine-triphosphate (UTP), the observed single turnover rate constants (*k*_obs_) for canonical nucleotide incorporation into the primer were compared for unmodified (A) and modified (m^6^A and Am) templates. *k*_obs_ values were measured for 0.1 μM RTC across a range of UTP concentrations (10–500 µM), permitting us to generate *K*_1/2_ curves for each template (Fig. 2A–C). Comparison of the maximum rate constants (*k*_max_) for transcription reveals that these two adenosine modifications slow transcription; *k*_max_ for the unmodified template is 900 ± 50 sec^−1^, while the m^6^A template has a fourfold lower *k*_max_ (220 ± 30 sec^−1^), and *k*_max_ for the Am template is reduced more than 500,000-fold (1.8 × 10^−3^ ± 0.1 × 10^−3^ sec^−1^) (Fig. 2D). Despite differences in *k*_max_, the *K*_1/2_ values did not change significantly when unmodified or modified templates were used, indicating that modified templates do not alter the UTP concentration dependence of SARS-CoV-2 transcription (Fig. 2E). These measurements together suggest that the efficiency of UTP addition by the SARS-CoV-2 RTC is lowered by transcribing modified adenosine templates, as the *k*_max_/*K*_1/2_ values for an unmodified template (12 ± 3 µM^−1^ sec^−1^) exceed those for the m^6^A (5 ± 3 µM^−1^ sec^−1^) and Am (3.1 × 10^−5^± 0.8 × 10^−5^ µM^−1^ sec^−1^) templates.

In addition, we observe that transcription on a template containing m^6^A in the +1 position has an end point defect relative to an unmodified template (Fig. 2F; Supplemental Fig. S3A). To assess if this results from the reduced assembly of the RTC on modified RNA templates, we performed electrophoretic mobility shift assays to establish the extent of complex assembly under our experimental conditions. We found that both templates equally bind the RTC proteins under our experimental conditions, indicating that the end point defect on m^6^A-containing templates does not arise from a failure of complex formation (Supplemental Fig. S4). Instead, this observation suggests that only a fraction of the complexes assembled on m^6^A templates are rapidly extended by the RTC (<1 sec), and that the remaining population of RTC may undergo a conformational change that allows templates to eventually be fully extended at longer timescales (Supplemental Fig. S3B). This idea is supported by the biphasic kinetics of elongation that we observe in extended time courses (Supplemental Fig. S3B). An increased propensity for the RTC to pause at modified sites has the potential to serve as a regulatory point for coronavirus transcription, such as allowing polymerase backtracking and proofreading by the viral 3′ exonuclease (ExoN) nsp14, which is part of the full RTC in vivo (Subissi et al. 2014).

**FIGURE 3.**
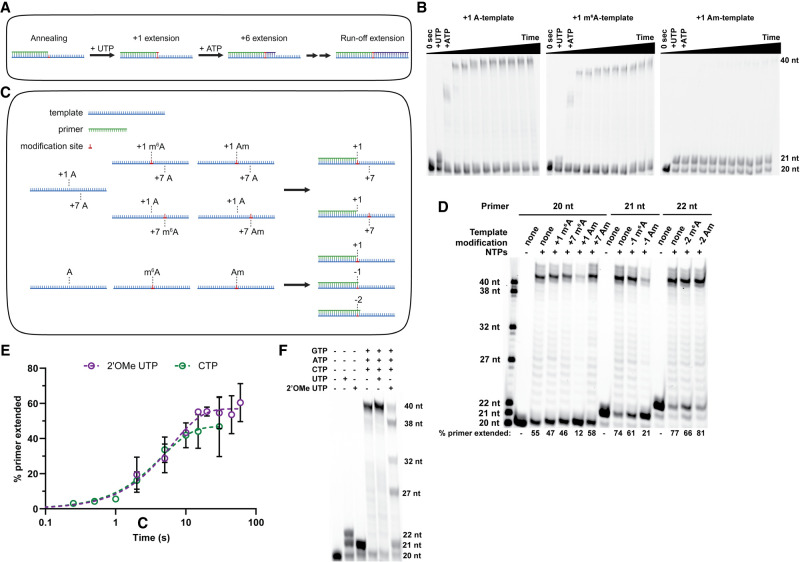
Modification location relative to transcription start site impacts RTC processivity. (*A*) Visual representation of double-mixing experiment performed in *B*, with RNA primer (green) annealed to RNA template (blue) being extended by the addition of UTP (red) and ATP (purple). (*B*) Time course of double-mixing experiment performed for the indicated template by the addition of UTP for 30 sec (A and m^6^A templates) or 1 h (Am template), followed by the addition of ATP up to 1 h. (*C*) Visual representation of RNA templates (blue) and RNA primers (green) used in *C*, showing modification sites relative to primer annealing and transcription start sites. (*D*) End point primer extension reactions performed by incubation of all NTPs with RNA duplexes composed of either unmodified, +1 m^6^A, +7 m^6^A, +1 Am, or +7 Am templates annealed to either a 20, 21, or 22 nt fluorescent primer for 5 min. Numbers *beneath* gel lanes represent the percentage of total RNA extension for a single reaction. Notably, when these reactions were run on a rapid quench, we observed the formation of 27 nt and longer products on unmodified and +7 m^6^A templates, but only up to 26 nt products on a +7 Am template, indicating that the RTC transiently pauses on Am under full elongation conditions. (*E*) Comparison of single-nucleotide addition kinetics for the addition of 2′OMe UTP or CTP using an unmodified template. (*F*) End point primer extension reactions performed by incubation of indicated NTPs with RNA duplexes composed of the unmodified template with 20 nt fluorescent primer for 10 min.

Our initial studies only evaluated primer extension by a single nucleotide. To investigate if modifications also impact the processivity of the RTC we next performed a double-mixing experiment. In this assay, we first extended the primer to the 21 nt product by adding ultrapure UTP, then subsequently added ATP to further extend the primer along the 5 nt downstream poly(U) sequence in the RNA template (Fig. 3A). Given this experimental setup, we expected to observe the primer being extended by 7 nt, with the extension stopping at the first G in the template. However, our RTC produced full-length 40 nt transcripts (Fig. 3B). This is in agreement with studies reporting that SARS-CoV-2 RTC has greater rates of nucleotide misincorporation in the presence of ATP (Pourfarjam et al. 2022); 98% and 97% of the initial 21 nt product generated upon UTP addition was extended using the unmodified and m^6^A templates after 5 min of incubation with ATP. In contrast, transcription using the Am template only achieved 12% further extension of the initial 21 nt product after 60 min of incubation with ATP, indicating that methylation of the 2′ hydroxyl (2′OH) on the template prevents translocation and stalls the polymerase at the modification site (Fig. 3B). These findings are consistent with a previous report finding that SARS-CoV-2 RdRp stalls after using Gm in the +2 position as a template (Petushkov et al. 2023).

We considered that the observed inhibition of transcription elongation may result from starting the primer extension reaction with the template modification already occupying the nsp12 active site. To assess this possibility, we examined the ability of the RTC to transcribe through modifications at a downstream starting position while maintaining the same primer and template annealing region, by using RNA templates modified at the +7 adenosine and initiating primer extension with a mixture of all canonical NTPs (Fig. 3C). The percentage of primer extension is similar for templates containing m^6^A at the +1 position (47%) or +7 position (46%). In contrast, primer extension on a +1 Am template (12%) is less than unmodified levels (55%), but restored when using a +7 Am template (58%) (Fig. 3D). While this experiment does not report on the rate of transcription, only the ability of the RTC to transcribe through the modified template, our results indicate that the RTC stalls less readily during productive elongation.

We also investigated how the proximity of a modified site upstream (3′) of the transcription start site impacted transcription processivity. To accomplish this, we used an experimental setup with 21 or 22 nt primers annealed over the modification to shift the transcription start site (Fig. 3D). The extent of primer extension on m^6^A-containing template remained consistent (61% with a 21 nt primer and 66% with a 22 nt primer) (Fig. 3D). The overall extension levels are slightly lower compared to an unmodified template (74% with a 21 nt primer and 78% with a 22 nt primer), as we would predict based on the decreased end point for m^6^A template in our single-nucleotide experiments (Fig. 2F; Supplemental Fig. S3A). These findings are in line with a previous observation that 20% of RNA elongated by the SARS-CoV-2 RdRp were truncated products when using templates containing +2 m^6^A modification, compared to 3% when using unmodified templates (Petushkov et al. 2023). Together, we conclude that the inclusion of m^6^A into template RNAs modestly slows the RdRp, although full-length transcription can still occur.

In contrast, we find that initiating primer extension downstream from the modification site can overcome stalls resulting from transcription on Am-containing templates, and that the efficiency of polymerase escape from stalling correlates with increasing the distance between the start site and the Am modification (21% with 21 nt primer vs. 81% with 22 nt primer) (Fig. 3D). Our results are generally consistent with past studies on flavivirus RdRps and more recent studies on SARS-CoV-2, which found that a poly(Am) template inhibits transcription (Dong et al. 2012; Jones et al. 2022). We posit that while 2′-*O*-methylation of template RNA cannot fully inhibit transcription in the presence of all NTPs, it greatly slows nucleotide incorporation and inhibits polymerase translocation when located near the transcription start site in agreement with studies on templates containing 2′-*O*-methylation in the +2 position (Petushkov et al. 2023). Internal 2′-*O*-methylation also has the potential to impact subgenomic mRNA generation if it is found in the transcription regulatory site (TRS), which is necessary for discontinuous transcription in coronaviruses (Zúñiga et al. 2004). However, coronaviruses encode many other nonstructural proteins that associate with the RTC in vivo, such as nsp13, an RNA helicase, and nsp14, a 3′–5′ exonuclease proofreader, that may also enable the RTC to overcome stalling (Subissi et al. 2014; Malone et al. 2021; Dangerfield and Johnson 2022).

Given our observation that the presence of Am in the template RNA significantly impacts RTC function, we considered the question of how SARS-CoV-2 RNA may come to contain 2′-*O*-methylated nucleotides. One possibility could be through the action of the SARS-CoV-2 encoded nsp16, a 2′-*O*-methyltransferase, which methylates the first and second nucleotides on the 5′ end of viral RNA to mimic eukaryotic cap structure, or from host 2′-*O*-methyltransferases (Krafcikova et al. 2020). Other viral nonstructural methyltransferases, such as flavivirus NS5, have been found to internally 2′-*O*-methylate viral RNA in addition to forming the eukaryotic cap 1 structure (Dong et al. 2012). A similar off-target effect of nsp16 may cause internal 2′-*O*-methylation of SARS-CoV-2 RNA, although coronavirus nsp12 and flavivirus NS5 have low sequence similarity overall (Krafcikova et al. 2020). In addition to being incorporated enzymatically, nonnative modifications can also be incorporated into viral RNA through the use of nucleotide analog inhibitors as therapeutics such as remdesivir (Tchesnokov et al. 2020). Regardless of how they are added, there is evidence that stalling effects elicited by these modifications can be overcome. For example, while remdesivir robustly inhibits SARS-CoV-2 transcription in vitro when low levels of NTPs are present, transcription stalling can be overcome by adding higher concentrations of NTPs. Notably, the “higher” concentrations are still well below the concentrations of NTPs found in cells (Tchesnokov et al. 2020).

Screens for nucleotide analog inhibitors identified many 1′ and 2′ modified nucleotides, including 2′-*O*-methylated nucleotide triphosphates, which were found to be delayed chain terminators of SARS-CoV-2 transcription (Jockusch et al. 2020; Yuan et al. 2021). We posited that the RTC might also be able to escape inhibition by these nucleotides, with the resulting transcripts reasonably used later in the viral life cycle as templates for RNA synthesis. To test this idea, we sought to examine how, and if, 2′-*O*-methylation of UTP impacts SARS-CoV-2 in vitro transcription. We began by using single turnover kinetics to determine the *k*_obs_ for single-nucleotide extension using an unmodified template with 500 µM of 2′-*O*-methylated UTP. We find that Um is incorporated 5000-fold slower than the canonical U (*k*_obs_ for 2′-*O*-methylated UTP = 0.16 ± 0.02 sec^−1^ compared to *k*_obs_ for 500 μM UTP = 800 ± 100 sec^−1^) (Fig. 3E). Notably, this rate defect is comparable to the single-nucleotide extension that we measured using the same unmodified template with 500 µM CTP (*k*_obs_ = 0.22 ± 0.04 sec^−1^). Our observation suggests that 2′-*O*-methylated NTPs could be incorporated at rates similar to those for the misincorporation of canonical nucleotides (Fig. 3E). By performing full primer extension assays using the unmodified template with UTP substituted for 2′OMe UTP, we also found that the RTC is capable of transcribing to the end of the template even after incorporating multiple 2′-*O*-methylated nucleotides (Fig. 3F). However, ∼10%–20% of primer extension reactions stalled at each adenosine in the template (Fig. 3F), in agreement with a study showing 2′OMe UTP is a partial chain terminator because it does not terminate transcription at a specific site (Yuan et al. 2021). Our results indicate further relevance for SARS-CoV-2 transcription inhibition through templated modifications.

**FIGURE 4.**
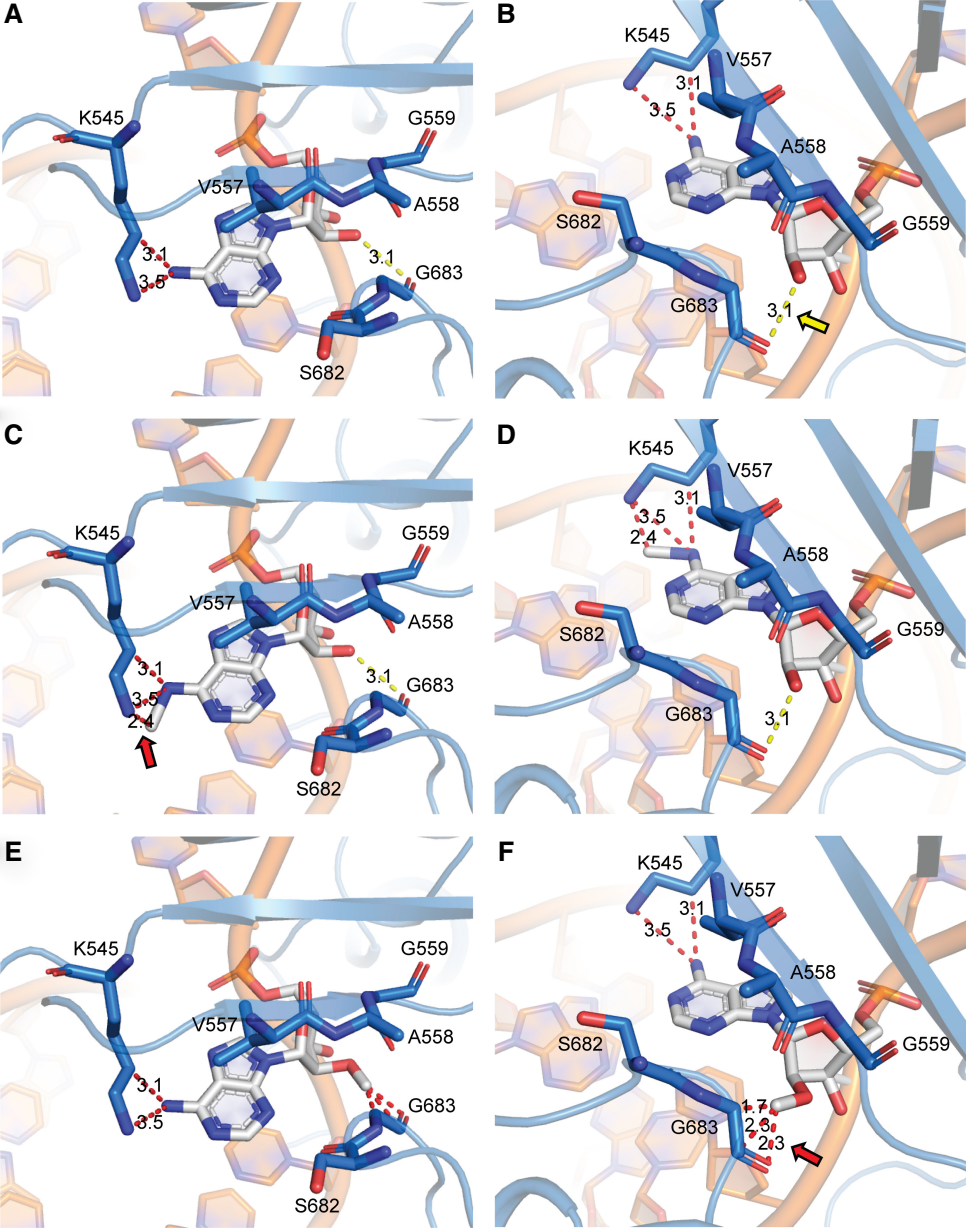
Model exploring the mechanistic basis for altered SARS-CoV-2 RdRp activity upon encountering methylation of adenosine in the +1 position (+1 A). The +1 A is shown with white sticks, RdRp amino acid residues proximal to the +1 nucleic acid residue are shown as blue sticks and labeled, hydrogen bonding interactions are shown with dashed yellow lines, and relevant distances are shown with a red dashed line. (*A*, *B*) The +1 A with no modifications. The +1 A 2′OH forms a hydrogen bond with the backbone of G683 (yellow arrow). (*C*, *D*) *N*^6^-methylation modestly increases the potential for steric clashes with K545 (red arrow). (*E*, *F*) 2′-*O*-methylation eliminates hydrogen bonding with G683 and introduces clashes (red arrow). Structures are based on PDB 6YYT (Hillen et al. 2020).

To gain structural insight into the positioning of modifications in the nsp12 active site, we examined the published structures of the minimal RTC (1 molecule of nsp12, 1 molecule of nsp7, 2 molecules of nsp8, template, and product RNA) and modeled m^6^A and Am into the +1 template position at the active site (Fig. 4; Hillen et al. 2020). In comparison to the unmodified template (Fig. 4A,B), the m^6^A *N*^6^-methyl group clashes with the sidechain of lysine 545 (Fig. 4C,D). K545 is part of the nsp12 finger domain and is responsible for the proper positioning of incoming nucleotides in the active site (Yin et al. 2020). The steric clashes introduced by m^6^A have the potential to disrupt the positioning of UTP in the active site and slow the rate of key conformational changes, potentially accounting for the observed end point defect in transcription assays we observed (Supplemental Fig. S3). Furthermore, in our model, the 2′-*O*-methyl group of Am exhibits multiple steric clashes and disrupts a hydrogen bond with the backbone of glycine 683 (Fig. 4E,F). Although interactions with RNA 2′OH groups typically serve to recognize RNA over DNA, G683 is part of the palm domain of nsp12, which contains the catalytic core of the enzyme (Yin et al. 2020). Collectively, our modeling and kinetic studies lead us to hypothesize that Am prevents the closure of nsp12 during catalysis through these interactions with the palm domain.

Our results demonstrate the impact of common adenosine methylations on the transcription activity of SARS-CoV-2 RdRp. While m^6^A has a modest effect, Am slows the rate of transcription by several orders of magnitude, likely changing the rate-limiting step of the reaction. These reductions in rate could be exploited by biology to introduce points of viral regulation. The *k*_obs_ of RNA degradation by nsp14 has been reported to be 2 sec^−1^, several orders of magnitude slower than the *k*_obs_ of nucleotide addition (Dangerfield and Johnson 2022). This raises the question of how nsp14 interacts with the rapidly elongating RNA product. Slowing of the RTC at a modification site on the template has the potential to increase both polymerase backtracking mediated by the nsp13 RNA helicase, and nsp14 3′–5′ exonuclease activity. Further understanding of the kinetics of SARS-CoV-2 genome maintenance, as well as the precise identity and location of modifications on the viral RNA, will provide potential druggable targets for the treatment of coronavirus infection.

## MATERIALS AND METHODS

### Nsp12 expression and purification

The plasmid for nsp12 protein expression (pRSFDuet-sumo- NSP12 [SARS-CoV-2]) was a gift from Thomas Tuschl (Addgene plasmid 159107; http://n2t.net/addgene:159107; RRID:Addgene 159107). The plasmid was transformed in BL21*(DE3) RIL codon plus competent cells (Agilent), and cells were grown in 6 L of LB media containing 50 µg/mL kanamycin and 34 µg/mL chloramphenicol at 37°C and 250 rpm. At OD_600_ 0.6, the cells were cooled on ice and induced by the addition of 0.1 mM IPTG, then grown at 16°C overnight. Cells were harvested by centrifugation at 5000 rpm for 20 min, then washed with lysis buffer (100 mM HEPES-KOH, 500 mM KCl, 5% glycerol, 2 mM MgCl_2_, 10 µM ZnCl_2_, 5 mM β-mercaptoethanol, 6 µM leupeptin, 20 µM pepstatin A, 20 µM benzamidine HCl hydrate, 10 µM PMSF). Cells were lysed using a homogenizer and then centrifuged at 16,000*g* at 4°C for 30 min. The soluble lysate was loaded on a 5 mL HisTrap column (Cytiva) equilibrated in Ni buffer A (20 mM HEPES-KOH, 300 mM KCl, 5% glycerol, 2 mM MgCl_2_, 10 µM ZnCl_2_, 5 mM β-mercaptoethanol, 6 µM leupeptin, 20 µM pepstatin A, 20 µM benzamidine HCl hydrate, 10 µM PMSF 30 mM imidazole). Elution from the HisTrap column was performed using Ni buffer B (20 mM HEPES-KOH, 10 mM KCl, 5% glycerol, 2 mM MgCl_2_, 10 µM ZnCl_2_, 5 mM β-mercaptoethanol, 500 mM imidazole). The elution was passed over a 6 mL Resource Q ion exchange column (Cytiva) equilibrated in Q buffer A (20 mM HEPES-KOH, 10 mM KCl, 5% glycerol, 2 mM MgCl_2_, 10 µM ZnCl_2_, 1 mM DTT). The flowthrough was mixed with Ulp1 protease while dialyzing overnight at 4°C against dialysis buffer (20 mM HEPES-KOH, 300 mM KCl, 5% glycerol, 2 mM MgCl_2_, 10 µM ZnCl_2_, 1 mM DTT). After dialysis, the protein was run through a 5 mL HisTrap column equilibrated in dialysis buffer; the flowthrough was collected and concentrated to ∼2.5 mL, then loaded on a HiLoad Superdex 200 column (Cytiva Life Sciences) equilibrated in SEC buffer (20 mM HEPES-KOH, 75 mM KCl, 5% glycerol, 5 mM MgCl_2_, 1 mM DTT). Final protein purity was assessed by SDS-PAGE, and fractions containing Nsp12 were concentrated and stored at −80°C.

### Nsp7 and Nsp8 expression and purification

Nsp7 and 8 genes were synthesized by Twist Bioscience and cloned into a pET-29b vector with a C-terminal 6xHix tag on Nsp8 and an N-terminal S-tag on Nsp7. Proteins were expressed in BL21*(DE3) competent cells and grown in 1 L TB media containing 50 µg/mL kanamycin at 37°C at 150 rpm. At OD_600_ 0.6, the cells were induced with 0.5 mM IPTG and grown overnight at 16°C. Cells were harvested by centrifugation at 5000 rpm for 20 min, then washed with lysis buffer (50 mM Tris-HCl, 300 mM NaCl, 10% glycerol, 5 mM MgCl_2_, 10 mM imidazole, 3 mM β-mercaptoethanol, 6 µM leupeptin, 20 µM pepstatin A, 20 µM benzamidine HCl hydrate, 10 µM PMSF). Cells were lysed by sonication, then centrifuged at 16,000*g* at 4°C for 30 min. The soluble lysate was loaded on a 5 mL HisTrap column (Cytiva) equilibrated in lysis buffer. Proteins were eluted from the HisTrap column with Ni elution buffer (50 mM Tris-HCl, 300 mM NaCl, 10% glycerol, 5 mM MgCl_2_, 500 mM imidazole, 3 mM β-mercaptoethanol, 6 µM leupeptin, 20 µM pepstatin A, 20 µM benzamidine HCl hydrate, 10 µM PMSF). Protein fractions were mixed with TEV protease and dialyzed against buffer (50 mM Tris-HCl, 300 mM NaCl, 5% glycerol, 5 mM MgCl_2_, 5 mM DTT) overnight at 4°C. Proteins flowed through a 6 mL Resource Q ion exchange column equilibrated in dialysis buffer were found in the flowthrough, which was then concentrated and loaded on a HiLoad Superdex 75 column (Cytiva Life Sciences) equilibrated in SEC buffer (50 mM Tris-HCl, 300 mM NaCl, 5% glycerol, 5 mM MgCl_2_, 1 mM DTT). Purity was assessed by SDS-PAGE, and fractions containing Nsp7 and 8 were concentrated and stored at −80°C.

### RNA design for transcription assays

RNA sequences are listed in Supplemental Table S1. All RNA was synthesized by Horizon Discovery; sequences used are listed in Supplemental Table S1. For primer extension assays, a 20, 21, or 22 nt primer with a fluorescein tag on the 5′ end was used for detection. A 40 nt template based on the 3′ end of SARS-CoV-2 RNA was used; the +1 adenosine was changed to the modified nucleoside of interest (Dangerfield et al. 2020). The +2 adenosine was changed to uridine to allow single-nucleotide extension with UTP. Further modified RNA templates were designed by moving the modification site to the +7 adenosine in the sequence.

### Single-nucleotide extension assays

RNA template and RNA primer were annealed in RNA buffer (20 mM HEPES-KOH, 50 mM KCl, 5 mM MgCl_2_) by heating to 80°C and slow cooling to 10°C on a thermocycler and then placed on ice. Nsp12 and nsp7 + 8 were mixed in protein buffer (20 mM HEPES-KOH, 50 mM KCl, 5 mM MgCl_2_, 5 mM DTT, 5% glycerol). RNA and protein were mixed and incubated at 37°C for 20 min. Reactions were initiated by the addition of a single ultrapure NTP in protein buffer. The final concentrations of the reaction were 0.1 µM primer RNA, 0.1 µM template RNA, 1 µM nsp12, 1 µM nsp7 + 8, and a titration of NTP concentration from 10 to 500 µM. Rapid quench flow reactions were quenched by the addition of 3 M HCl to a final concentration of 1 M HCl. Samples were ethanol precipitated, then resuspended in gel loading buffer containing 49% formamide, 5 mM EDTA, and bromophenol blue. Benchtop assays were quenched by pipetting the reaction mixture into tubes containing 2× stop buffer (8 M urea, 10 mM EDTA, 2× TBE, and bromophenol blue). All samples were run on 20% Urea-PAGE gels. Gels were scanned on a Typhoon spectrometer to visualize the fluorescein-tagged primer and extension products. Bands were quantified using ImageQuant (Cytiva Life Sciences) and fit to pseudo-first-order kinetic equations using Prism (GraphPad Software). Time course data were fit using Equation 1:

$$\mathrm{Fraction}\;\mathrm{product}\;=A\cdot (\;1-e^{-k_{\mathrm{obs}}t})$$

*K*_1/2_ curves were fit using Equation 2:

$$k_{\mathrm{obs}}=\frac{k_{\max}\;*\;[\mathrm{UTP}]}{K_{1/2}+[\mathrm{UTP}]}$$

### Structure analysis

The structure of SARS-Cov-2 RdRp in complex with an RNA template containing the antiviral remdesivir (PDB:6YYT) (Hillen et al. 2020) was modified at the +1 base position from a guanidine to an adenine or an m^6^A using Coot (version 0.9.6) (Emsley et al. 2010). To ensure a structurally relevant orientation of the 2′-*O*-methylation, a structure of a 2′-*O*-methylated ribose from a modified RNA base in a helical and base-stacking orientation comparable to the ribose orientation present in 6YYT (PDB: 1I7J) (Adamiak et al. 2001) was used to replace the +1 ribose base in our model. Interactions were analyzed and the model was visualized using PyMol (version 2.5.7).

## SUPPLEMENTAL MATERIAL

Supplemental material is available for this article.

## Figures and Tables

**Laura R. Snyder:**
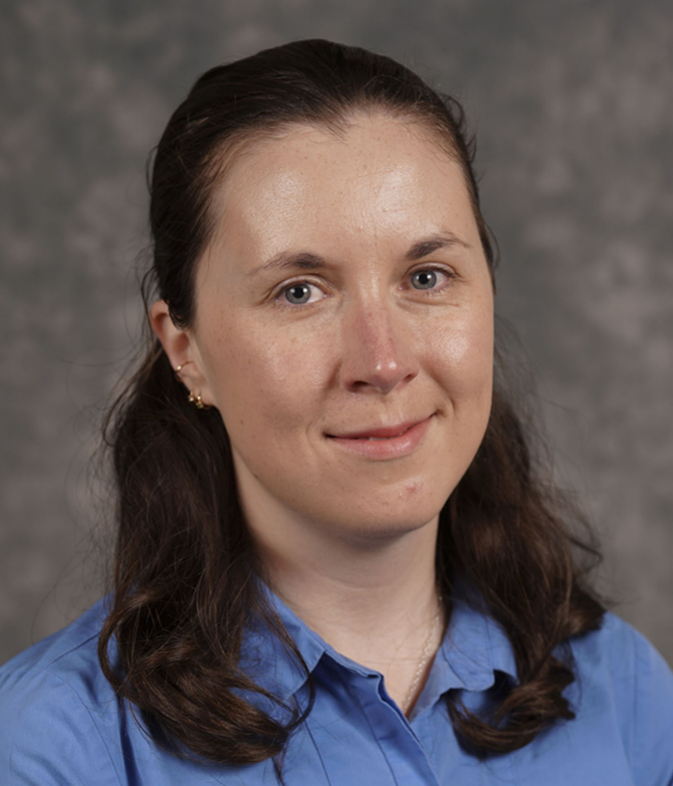


## MEET THE FIRST AUTHOR

***Meet the First Author(s)* is an editorial feature within *RNA*, in which the first author(s) of research-based papers in each issue have the opportunity to introduce themselves and their work to readers of *RNA* and the RNA research community. Laura R. Snyder is the first author of this paper, “Adenosine modifications impede SARS-CoV-2 RNA-dependent RNA transcription.” Laura is a fifth-year PhD student in the lab of Kristin Koutmou at the University of Michigan Department of Chemistry. The main focus of her research is how RNA modifications impact the RNA replication machinery of the virus SARS-CoV-2.**

**What are the major results described in your paper and how do they impact this branch of the field?**

The major results from my paper are that the adenosine modifications *N*^6^-methyladenosine and 2′-*O*-methyladenosine can slow the rate of single-nucleotide addition by the SARS-CoV-2 RNA-dependent RNA polymerase, although they do not stop the polymerase entirely. My results add data on single-nucleotide kinetics to a growing body of research about the impact of modifications on SARS-CoV-2 transcription. RNA replication is an important step in the viral life cycle, and time-resolved data helps us understand the dynamics of viral RNA polymerases under different conditions, such as the presence of RNA modifications.

**What led you to study RNA or this aspect of RNA science?**

I joined the Koutmou lab at the University of Michigan in 2020. I already knew I would be studying RNA modifications but we had not yet decided on a specific project, although I had worked on modifications in MS2 bacteriophage during my fall rotation. Therefore, my growing interest in the effect of RNA modifications on RNA viruses coincided with the beginning of the COVID-19 pandemic, which lead to an entirely new project in the Koutmou lab.

**During the course of these experiments, were there any surprising results or particular difficulties that altered your thinking and subsequent focus?**

One particular difficulty was the purification of the SARS-CoV-2 protein Nsp12, the key protein for my in vitro transcription assays. I encountered many roadblocks during expression and purification, including insoluble protein, low yields, and initially slower enzyme kinetics than expected for this polymerase based on other publications. All of these issues required careful troubleshooting and optimization, which was time-consuming but also helped me develop more independence and patience in the laboratory.

**What are some of the landmark moments that provoked your interest in science or your development as a scientist?**

Some of the landmark moments that steered me toward a career in science were the science elective classes I was able to take in high school. In particular, I chose to take a Saturday organic chemistry class and an introductory genetics class. I had great teachers who worked hard to show their students how awesome and fun science is. Considering how formative that was for me, I feel it is really important to expand similar education opportunities to as many young people as possible.

**What are your subsequent near- or long-term career plans?**

I plan to defend my thesis and graduate in fall 2024. Afterward, I want to return to the east coast of the USA near Philadelphia, where I am from, and continue as a scientist in the biotech or pharmaceutical industries. I hope to continue working on RNA wherever I am!
